# Mechanism of Reduced Sintering Temperature of Al_2_O_3_–ZrO_2_ Nanocomposites Obtained by Microwave Hydrothermal Synthesis

**DOI:** 10.3390/ma11050829

**Published:** 2018-05-17

**Authors:** Iwona Koltsov, Julita Smalc-Koziorowska, Marta Prześniak-Welenc, Maria Małysa, Giora Kimmel, Jessica McGlynn, Alexey Ganin, Swietlana Stelmakh

**Affiliations:** 1 Institute of High Pressure Physics, Polish Academy of Sciences, Sokolowska 29/37, 01-142 Warsaw, Poland; julita.smalc.koziorowska@unipress.waw.pl (J.S.-K.); malysa.maria@gmail.com (M.M.); svetlana.stelmakh@unipress.waw.pl (S.S.); 2 Faculty of Applied Physics and Mathematics, Gdansk University of Technology, Narutowicza 11/12, 80-233 Gdansk, Poland; mprzesniak@mif.pg.gda.pl; 3 Department of Nuclear Engineering, Ben-Gurion University of the Negev, Beer Sheva 8410501, Israel; kimmel@exchange.bgu.ac.il; 4 WestCHEM, School of Chemistry, University of Glasgow, University Avenue, Glasgow G12 8QQ, UK; j.mcglynn.1@research.gla.ac.uk (J.M.); alexey.ganin@glasgow.ac.uk (A.G.)

**Keywords:** microwave hydrothermal synthesis, Al_2_O_3_–ZrO_2_ nanocomposites, shrinkage temperature, grain boundaries, isolation effect of t-ZrO_2_, phase composition

## Abstract

A novel method to obtain Al_2_O_3_–ZrO_2_ nanocomposites is presented. It consists of the co-precipitation step of boehmite (AlO(OH)) and ZrO_2_, followed by microwave hydrothermal treatment at 270 °C and 60 MPa, and by calcination at 600 °C. Using this method, we obtained two nanocomposites: Al_2_O_3_–20 wt % ZrO_2_ and Al_2_O_3_–40 wt % ZrO_2_. Nanocomposites were characterized by Fourier transformed infrared spectroscopy, Raman spectroscopy, X-ray diffraction, and transmission electron microscopy. Sintering behavior and thermal expansion coefficients were investigated during dilatometric tests. The sintering temperatures of the nanocomposites were 1209 °C and 1231 °C, respectively—approximately 100 °C lower than reported for such composites. We attribute the decrease of the sintering temperature to the specific nanostructure obtained using microwave hydrothermal treatment instead of conventional calcination. Microwave hydrothermal treatment resulted in a fine distribution of intermixed highly crystalline nanoparticles of boehmite and zirconia. Such intermixing prevented particle growth, which is a factor reducing sintering temperature. Further, due to reduced grain growth, stability of the θ-Al_2_O_3_ phase was extended up to 1200 °C, which enhances the sintering process as well. For the Al_2_O_3_–20 wt % ZrO_2_ composition, we observed stability of the zirconia tetragonal phase up to 1400 °C. We associate this stability with the mutual separation of zirconia nanoparticles in the alumina matrix.

## 1. Introduction

Alumina-toughened zirconia (ATZ) and zirconia-toughened alumina (ZTA) are important materials for high-temperature structural [1,2,3] and medical [4,5,6,7] applications due to their excellent strength and toughness, high wear resistance and temperature stability, and the fact that they are both chemically inert. In order to obtain these properties, the tetragonal metastable phase of zirconia (t-ZrO_2_) needs to remain stable at room temperature; however, t-ZrO_2_ is normally only stable at temperatures above 1170 °C. One method for ensuring that t-ZrO_2_ remains stable at room temperature is to partially stabilize it with an yttria (Y_2_O_3_) dopant (YSZ) [4,5,6,8,9].

In general, there are two ways to stabilize t-ZrO_2_ particles: the first is by alloying them with other materials while the other is by ensuring that their particle size does not exceed 35 nm. Stabilization of the t-ZrO_2_ phase requires certain special synthesis techniques to be used, especially when one desires to have a uniform zirconia particle distribution in an alumina matrix [10,11]. Recent work has shown that synthesizing Al_2_O_3_ with ZrO_2_ nanopowders (up to ~40 wt % ZrO_2_) could lead to the partial stabilization of t-ZrO_2_ [12].

In ZTA composites, t-ZrO_2_ nanoparticles are stable at room temperature; however, under stress, these nanoparticles may undergo a transformation from the tetragonal to the monoclinic phase [8,13,14]. More specifically, if cracks were to propagate through such a ceramic, then any t-ZrO_2_ particles in the region of the crack could potentially undergo a martensitic transformation from the tetragonal (t-ZrO_2_) to the monoclinic phase (m-ZrO_2_); a volume expansion of about 3% would also occur. This would generate compressive stresses in the alumina matrix, preventing the crack from increasing in size and causing the fracture toughness of the ceramic to increase [15,16].

Various chemical methods have so far been used to synthesize Al_2_O_3_–ZrO_2_ and YSZ/Al_2_O_3_ ceramics, such as the sol-gel method [17], hydrothermal synthesis followed by calcination [18], and the co-precipitation method [19]. Another method, the microwave hydrothermal synthesis method (MHS), has been found to be particularly attractive due to its potential to produce highly crystalline nanoparticles with a narrow particle size distribution [20,21].

Microwave hydrothermal synthesis (MHS) is a method which possesses many advantages in comparison to standard heating methods [20,21,22]. Firstly, the MHS method is characterized by considerably shorter reaction time. In very short time, the reaction vessel is heated up rapidly and uniformly and the precipitate is heated directly through the energy of microwaves. As a result, heat is generated within the whole volume of the sample and not transported through the reaction vessel walls from an external heat source [22]. Secondly, thanks to uniform heating, the crystallization process in the sample is quick and uniform, resulting in a narrow and nanometric particle size distribution [21,22,23,24]. Another advantage is that reaction vessels used in MHS do not introduce contaminants by the contactless heating method. In addition, the low thermal conductivity (0.25 W/(m K)) of Teflon^®^ causes a small temperature gradient between a sample and a vessel which contributes to the uniformity of the product [21]. The biggest advantage of microwave synthesis over other synthesis methods is that it delivers energy directly to the substance without thermal-conductivity-related constraints [22].

Microwave hydrothermal synthesis methods can be used to produce nanomaterials with a variety of morphologies and programmed sizes. Variations in these attributes can significantly affect the properties of final products [25]. Furthermore, using MHS for the production of ZrO_2_–Al_2_O_3_ nanopowders may lead to the isolation of t-ZrO_2_ in the alumina matrix in these materials, and thus to the reduction of aggregation.

ZrO_2_–Al_2_O_3_ nanocomposites are often obtained by the sintering of compactified powders [11,26,27,28,29]. The microstructure of a ceramic depends upon the structure of the initial materials used for its synthesis, the dopant distribution used, the heating rate, and the sintering mechanism used. For the manufacture of Al_2_O_3_–ZrO_2_ ceramics, it is crucial that a suitable sintering temperature is chosen. One study that illustrates this was conducted by Zhuravlev et al. [30], who showed that the maximum density of Al_2_O_3_ ceramics with partially stabilized ZrO_2_ (3YSZ) was achieved during sintering at 1400–1500 °C. The density of composite decreased and the open porosity of the ceramic materials grew with increasing amount of Al_2_O_3_. Several studies have manufactured dense ceramics while maintaining a small grain size; most of them used optimized sintering strategies or advanced sintering techniques [11,26,31]. Numerous analyses of the mechanical properties of Al_2_O_3_–ZrO_2_ composite systems have also been reported upon [32,33,34,35,36,37], and a wide range of results have been presented. These results were influenced by the fabrication routes used, the composition design, and the measurement technique used.

However, there is a lack of data in the literature about the sintering behavior of Al_2_O_3_–ZrO_2_ nanocomposites obtained by the method involving co-precipitation and subsequent MHS. Co-precipitation leads to the uniform distribution of components which is very difficult to achieve when mixing the two powders. MHS, meanwhile, leads to the creation of fully crystalline particles (which are not synthesized in the sol-gel process). The sintering of fully crystalline particles may lead to ceramic structures being created than could not be obtained by the sintering of sol-gel-precipitated particles.

The aim of the work was to develop a new method for the manufacturing of Al_2_O_3_–ZrO_2_ nanocomposites with stable t-ZrO_2_ and with reduced sintering temperature. The present work reports a series of experiments for finely grained Al_2_O_3_–ZrO_2_ nanocomposites with 20 and 40 wt % ZrO_2_. Nanocomposites were obtained by co-precipitation and MHS before being thermally treated. We discuss the roles that chemical composition, morphology, and phase composition had in reducing the sintering temperature used for the synthesis of nanocomposites to levels significantly below that found in the literature for Al_2_O_3_–3YSZ and Al_2_O_3_–ZrO_2_.

## 2. Materials and Methods

The procedure for synthesizing nanopowders containing boehmite (AlO(OH)) with 20 and 40 wt % ZrO_2_ is described in detail elsewhere [12]. The reagents used in the process were zirconyl chloride octahydrate (ZrOCl_2_·8H_2_O, Sigma-Aldrich (≥99.5%)), sodium hydroxide (CHEMPUR, analytically pure), and aluminium nitrate nonahydrate (Al(NO_3_)_3_·9H_2_O, CHEMPUR, analytically pure). Microwave reactions took place in a MAGNUM II ERTEC microwave reactor (2.45 GHz, 600 W). The synthesis of a homogenous mixture of Al_2_O_3_ and ZrO_2_ nanocomposites took place in four steps:

- The first was the co-precipitation of the precursors;
- The second was 20 min of MHS at *t* = 270 °C and *p* = 60 atm, in order to obtain a crystalline mixture of AlO(OH) and ZrO_2_;
- The third was the drying of the precipitates at room temperature. The mixture of nanopowders obtained in step three will be called AlO(OH)–ZrO_2_ or as-synthesized;
- The fourth step was calcination of nanopowder mixture at 600 °C for 2 h in order to obtain ZrO_2_ with γ-Al_2_O_3_ originated from AlO(OH).

Following the above-described four-step procedure, a homogenous mixture of Al_2_O_3_ and ZrO_2_ nanopowders was produced. For the characterization experiments carried out in this work, some of the calcinated powders were also conventionally sintered in air at 1200, 1400 and 1500 °C for 1 h.

Samples were prepared for inductively coupled plasma optical emission spectroscopy (ICP-OES, Perkin-Elmer, Optima 8300, Waltham, MA, USA) in a microwave mineralizer (AntonPaar, MW 3000, Graz, Austria) in a Teflon vial using a mixture of acids, namely, HF/HNO_3_ and H_2_SO_4_. The ICP-OES tests were conducted according to the standard procedure described in EN ISO 11885:2009 [38].

Before and after heating, samples were examined using a Fourier transform infrared (FTIR) spectrometer (Bruker Optics, Tensor 27, Bruker BioSpin GmbH, Rheinstetten, Germany) equipped with a diamond attenuated total reflectance (ATR) accessory. The diamond ATR crystal was soldered onto a ceramic plate; this resulted in the sampling area being chemically inert and mechanically rugged. All of the ATR-FTIR spectra were recorded at room temperature in the 4000–400 cm^−1^ range. The spectral resolution and accuracy of the measurements were ±4 cm^−1^ and ±1 cm^−1^, respectively.

A Raman scattering spectroscope (Horiba-Jobin-Yvon, LabRam, HR800, Kyoto, Japan) operated with a λ = 532 nm laser was used to investigate samples annealed at 600 and 1400 °C. In order to prevent the samples from degrading, a 10% filter was used. Additionally, an aperture size of 100 μm was used.

X-ray diffraction (XRD) patterns of the nanopowders were collected on a diffractometer (X’Pert PRO, PANalytical, Almelo, Netherlands) equipped with a copper anode (Cu Kα1) and an ultra-fast PIXcel1D detector. The analysis was performed at room temperature in the 2θ range of 10–100° with a step size of 0.03°. All of the diffraction data was analyzed using advanced data processing methods, including Rietveld refinement [39], by the programs DBWS [40] and FullProf [41]; these were used for the structural and quantitative phase analyses, respectively. The peak shape function used in calculation was pseudo-voigt.

The surface morphology was evaluated using a scanning electron microscope (Zeiss, Ultra Plus, Zeiss, Oberkochen, Germany), and the compositions of the nanopowders were estimated semiquantitatively using energy dispersive spectroscopy (EDS) (QUANTAX 400, Bruker, Billerica, MA, USA). For this evaluation, the synthesized materials were compacted to pellets and sintered at 1300 °C for 1 h. Measurements were done as per the ISO 22309:2011 standard [42].

The microstructures of the nanopowders and nanocomposites were investigated using conventional high-resolution (HR) transmission electron microscopy (TEM) and scanning (STEM) techniques with a FEI TECNAI G2 F20 S-TWIN electron microscope (Thermo Fisher Scientific, Waltham, MA, USA).

Helium density measurements were carried out using a helium pycnometer (AccuPyc II 1340, FoamPyc V1.06, Micromeritics, Norcross, GA, USA). The measurements were carried out in accordance with the ISO 12154:2014 standard [43] at temperatures of 25 ± 2 °C.

The specific surface area (SSA) of the nanopowders was determined in accordance with ISO 9277:2010 [44]; more specifically, a surface analyzer (Gemini 2360, V 2.01, Norcross, GA, USA) that used the nitrogen adsorption method was used, and this method was based on the linear form of the Brunauer–Emmett–Teller (BET) isotherm equation. The detailed experimental procedure and the determination of particle size using the BET method (SSA_BET_) is described elsewhere [12].

In order to calculate the crystallite diameter and size distribution, equations dedicated to spherical crystallites in the Nanopowder XRD Processor Demo (Pielaszek Research) web application were used. The website provides an online tool in which diffraction files can be directly uploaded [45]; the files are then processed on a server and the particle size distribution for the XRD peaks is extracted.

The nanopowders calcinated at 600 °C were compacted at room temperature in a stainless steel mold under 250 bar. Dilatometric experiments were also carried out; for these, cylindrical specimens with 5 mm diameters and ca. 5 mm heights were prepared. The shrinkage behavior of bulk samples and their relative changes in length were examined using a Netzsch DIL 402 PC/4 dilatometer in an argon ambient atmosphere between 40 and 1500 °C at a constant heating and cooling rate of 2 °C min^−1^. The densification rate was obtained by differentiating the measured linear shrinkage data. The temperature at which a change in the slope of the dilatometric relative length curve occurred was taken as being the phase transition temperature.

## 3. Results

**Figure 1:**
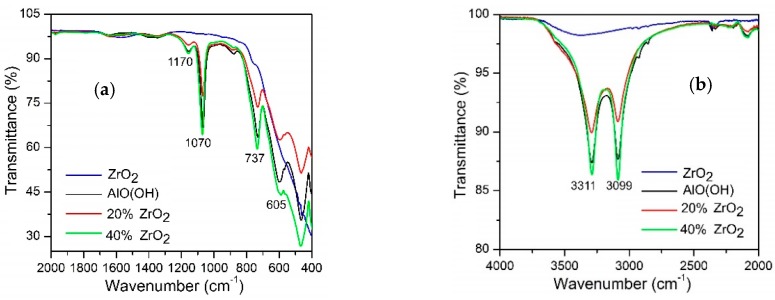

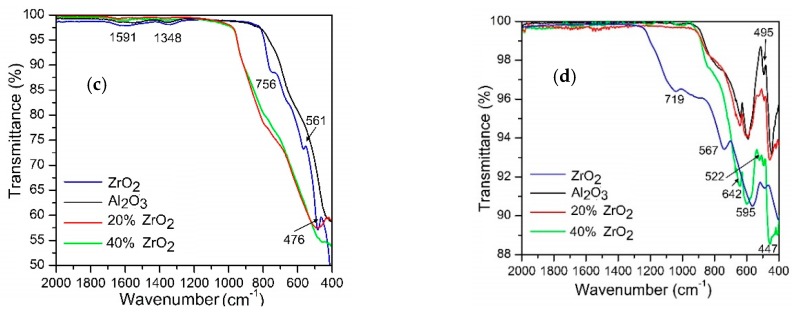
FTIR spectra for Al_2_O_3_–(20,40 wt %) ZrO_2_ in as-synthesized form (**a**,**b**), calcined at 600 °C (**c**), and after annealing at 1400 °C (**d**).

Figure 1 shows the FTIR spectra for (a), (b) as-synthesized AlO(OH)–ZrO_2_ nanopowders, (c) nanopowders after calcination at 600 °C, and (d) after annealing at 1400 °C. Pure ZrO_2_, boehmite (AlO(OH)), and Al_2_O_3_ were added as references. The bands observed for the as-synthesized samples were identified; they are listed in Table 1 [10,36,37,46,47,48,49,50,51,52].

**Figure 2:**
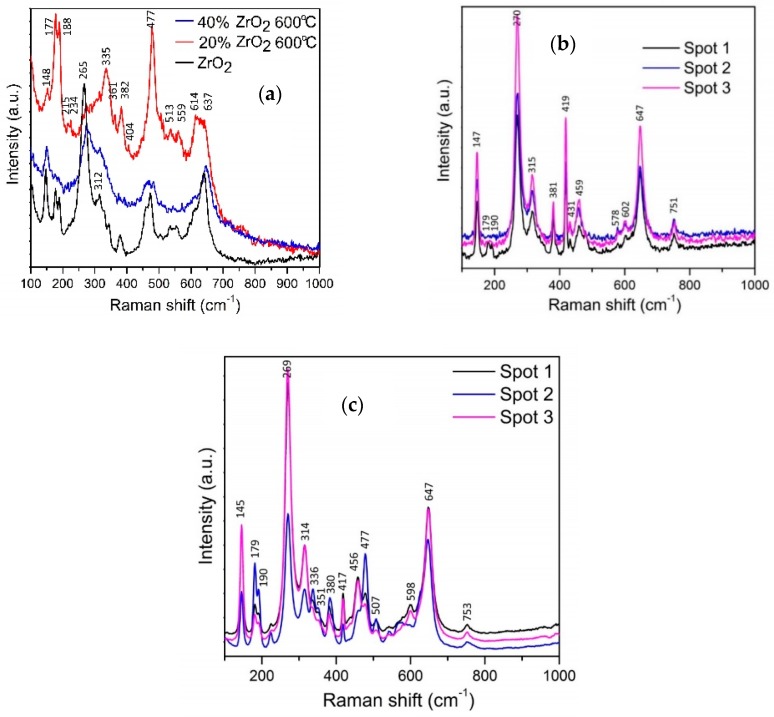
Raman spectra for (**a**) Al_2_O_3_–(20,40 wt %) ZrO_2_ after annealing at 600 °C, (**b**) Al_2_O_3_–20 wt % ZrO_2_ after annealing at 1400 °C, and (**c**) Al_2_O_3_–40 wt % ZrO_2_ after annealing at 1400 °C.

Figure 2 shows Raman spectra for Al_2_O_3_–ZrO_2_ nanocomposites calcinated at 600 °C and for pellets sintered at 1400 °C. The relevant peaks reported in the literature for t-ZrO_2_, m-ZrO_2_, and Al_2_O_3_ are listed in Table 2.

**Figure 3:**
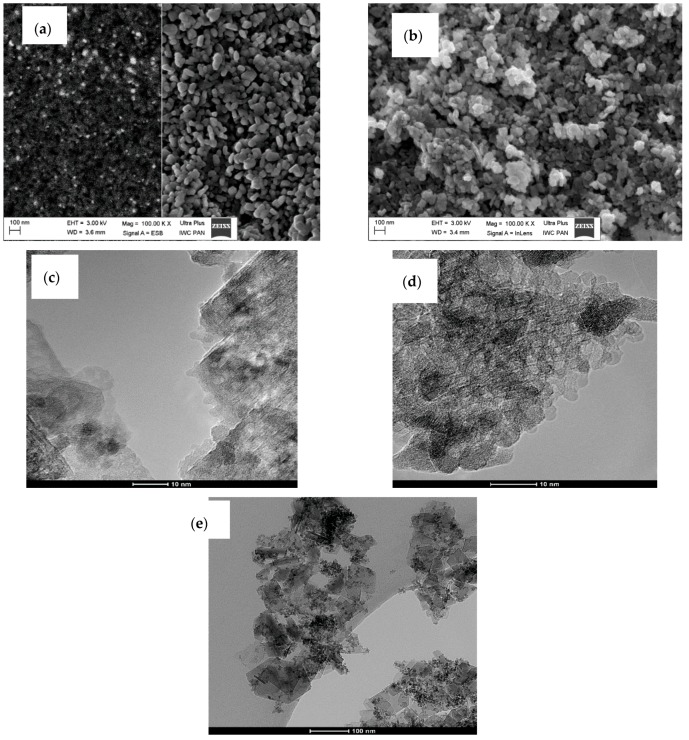
SEM and TEM images for Al_2_O_3_–20 wt % ZrO_2_ after synthesis (**a**,**c**), and Al_2_O_3_ with 40 wt % ZrO_2_ after synthesis (**b**,**d**,**e**).

**Figure 4:**
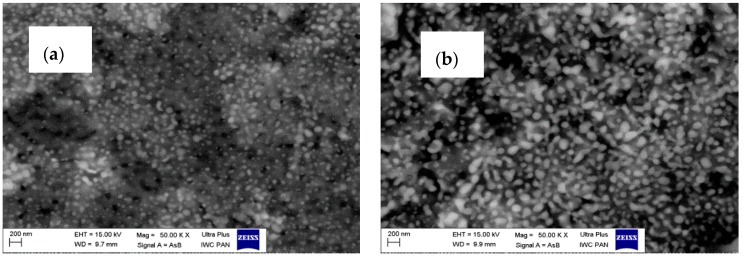

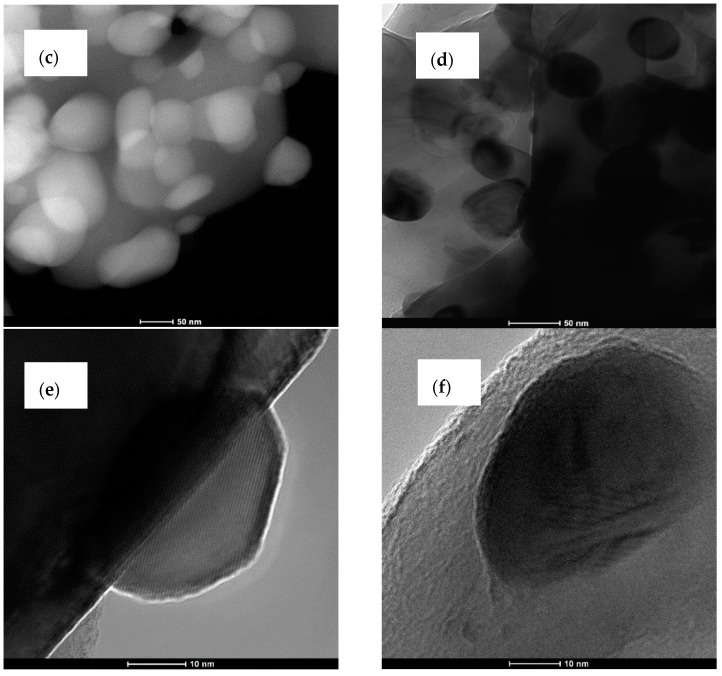
SEM and TEM images for Al_2_O_3_–20 wt % ZrO_2_ after annealing at 1400 °C (**a**,**c**,**e**), and Al_2_O_3_–40 wt % ZrO_2_ after annealing at 1400 °C (**b**,**d**,**f**).

The morphology of as-synthesized AlO(OH)–ZrO_2_ nanopowders is presented in Figure 3. There is very little difference between samples in terms of shape and particle size. In addition, Figure 4 shows TEM and SEM images of morphology and structure for nanocomposites obtained after annealing at 1400 °C.

**Figure 5:**
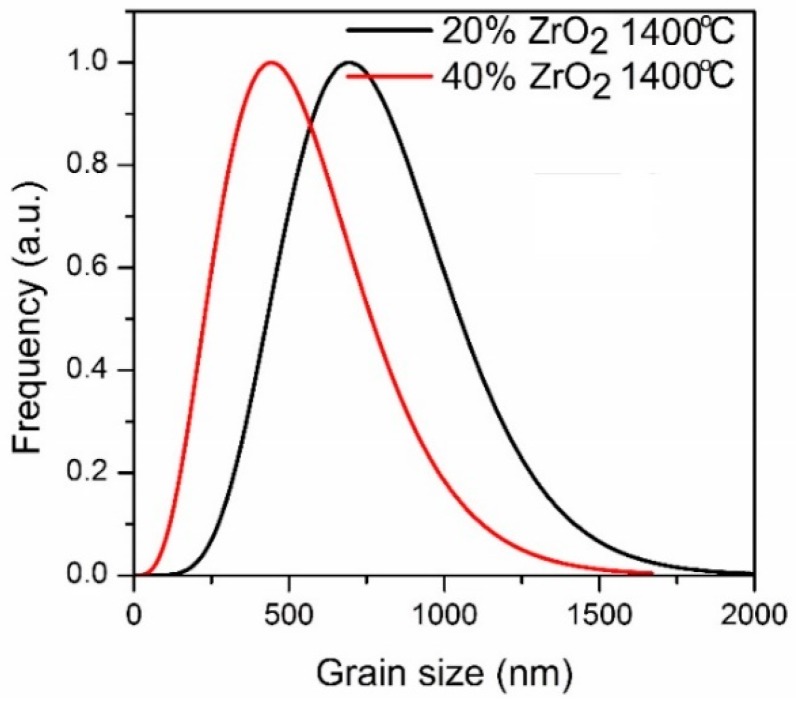
Particle size distribution for Al_2_O_3_–(20,40 wt %) ZrO_2_ nanocomposites after annealing at 1400 °C.

Particle size distributions for ZrO_2_ phases (both t-ZrO_2_ and m-ZrO_2_) are shown in Figure 5. Calculation was prepared based on XRD experiments.

The results for SSA_BET_ and density of nanopowders after calcination at 600 °C and 1400 °C are shown in Table 3. For both compositions there is visible difference in SSA_BET_ values after annealing at 600 °C: 177.2 for Al_2_O_3_–20 wt % ZrO_2_ and 95.9 m^2^/g for Al_2_O_3_–40 wt % ZrO_2_. The density values are comparable (3.5 g/cm^3^ for Al_2_O_3_–20 wt % ZrO_2_ and 3.8 g/cm^3^ for Al_2_O_3_–40 wt % ZrO_2_).

**Figure 6:**
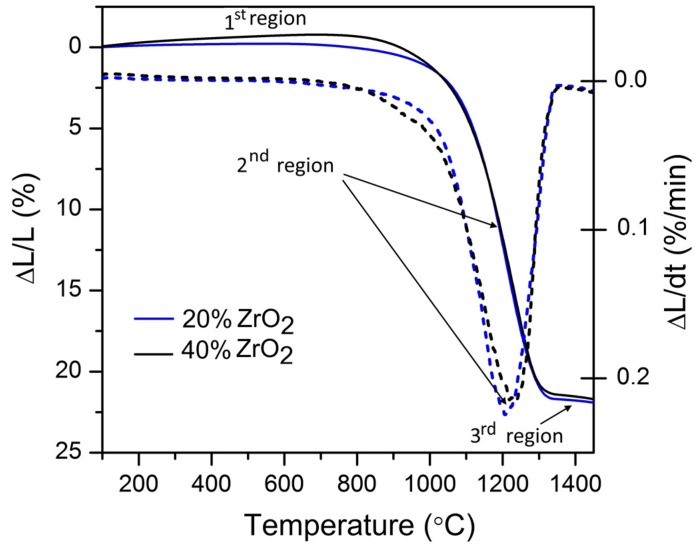
Dilatometric curves for Al_2_O_3_–(20,40 wt %) ZrO_2_ nanocomposites (solid lines), where dotted lines represent first derivative.

Figure 6 shows dilatometry curves for Al_2_O_3_–20 wt % ZrO_2_ and Al_2_O_3_–40 wt % ZrO_2_ with three distinguished regions characteristic of the sintering process.

In Table 4 we show results of chemical and phase composition of Al_2_O_3_–(20,40 wt %) ZrO_2_ nanocomposites annealed in the 600–1400 °C temperature range. These results were obtained from ICP-OES, EDS, and Rietveld refinement analysis.

**Figure 7:**
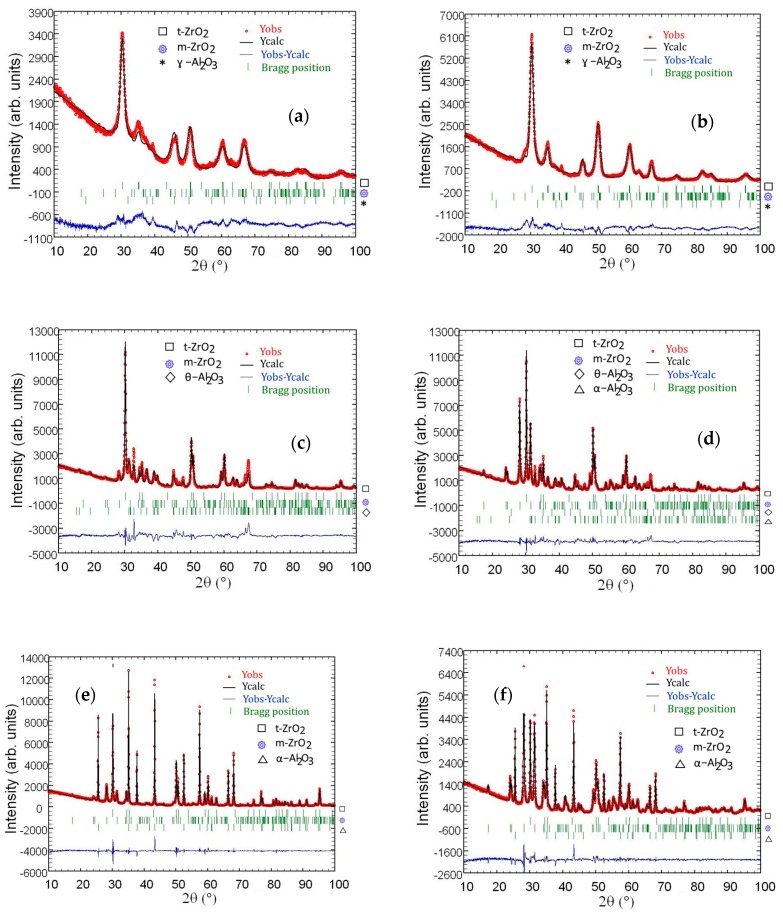
Rietveld refinement for Al_2_O_3_–20 wt % ZrO_2_ after annealing at 600 °C for 2 h (**a**), 1200 °C (**c**), 1400 °C (**e**), and Al_2_O_3_–40 wt % of ZrO_2_ after annealing at 600 °C for 2 h (**b**), 1200 °C (**d**), 1400 °C (**f**).

Rietveld refinements for Al_2_O_3_–(20,40 wt %) ZrO_2_ nanocomposites are presented in Figure 7. Refinement was performed for nanocomposites after annealing at 600 °C, 1200 °C and 1400 °C. Peak positions coming from analyzed phases are marked on the figures. Atom positions in analyzed phases as well as cell parameters are presented in Table 5 and Table 6.

**Figure 8:**
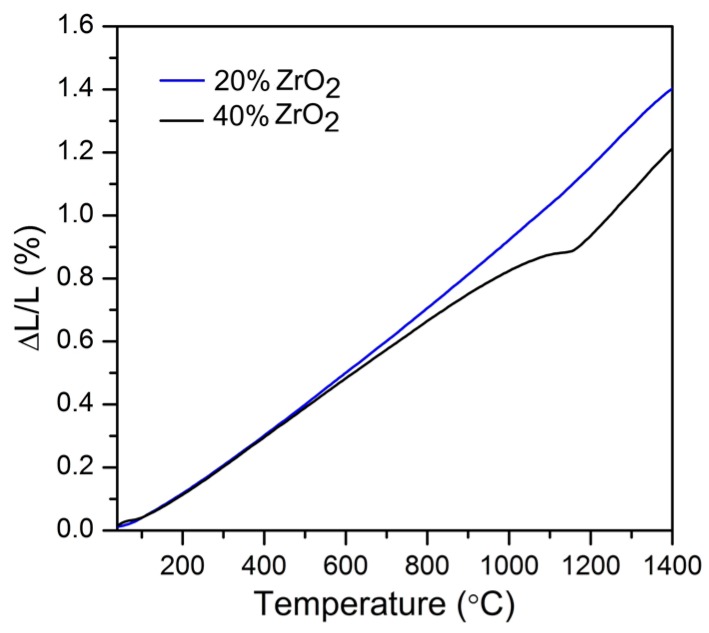
Shrinkage behavior of Al_2_O_3_–(20,40 wt %) ZrO_2_ nanocomposites.

The thermal parameters obtained from dilatometric experiments (including sintering temperature calculated using maximum shrinkage rate) are shown in Table 7. The thermal expansion coefficients were obtained by fitting the relative elongation as a function of temperature with linear functions (Figure 8).

## 4. Discussion

FTIR spectra show that there were signals associated with Al–O–H; these bands are characterized by bands in the OH– and water regions at 3311 and 3309 cm^−1^, respectively, in Figure 1a as well as by bands originating from Al–O in Figure 1b. In the figure, it can be seen that for Al_2_O_3_–ZrO_2_ (20 and 40 wt %) which are calcinated at 600 °C (Figure 1b), the only well-resolved peak that can be associated with ZrO_2_ is that of a broad shoulder that has a maximum at 476 cm^−1^, which can be assigned to t-ZrO_2_. Following calcination at 600 °C, alumina exists in the form γ-Al_2_O_3_; this is due to a boehmite transformation. Following annealing conducted at 1400 °C, the materials can be characterized by bands originating from Al_2_O_3_.

Raman results indicate that all compositions calcinated at 600 °C contained both moclinic and tetragonal zirconia phases. The amount of m-ZrO_2_ is significantly lower for Al_2_O_3_–40 wt % ZrO_2_ compared to Al_2_O_3_–20 wt % ZrO_2_ samples (Figure 2a). Furthermore, the Al_2_O_3_–40 wt % ZrO_2_ composite contains a significantly higher amount of t-ZrO_2_ than a pure ZrO_2_ sample (e.g., without any addition of Al_2_O_3_). These observations are in agreement with XRD results described in further paragraphs.

Annealing at 1400 °C of the Al_2_O_3_–20 wt %ZrO_2_ composition demonstrates that the t-ZrO_2_ phase dominates over the monoclinic phase and that the sample is homogeneous (Figure 2c). On the other hand, the composition of Al_2_O_3_–40 wt % ZrO_2_ shows that ZrO_2_ particles are mostly in the monoclinic phase (Figure 2c). Further analysis of the sample at several locations revealed that the sample is not homogeneous with some parts containing a considerable amount of t-ZrO_2_. It is possible to draw a conclusion that a higher content of ZrO_2_ leads to greater presence of m-ZrO_2_. Two pronounced peaks at 177 and 188 cm^−1^ indicate that Al_2_O_3_ has a stabilizing effect on t-ZrO_2_ in the case of the Al_2_O_3_–20 wt % ZrO_2_ composition.

Composites present two types of morphologies: boehmite thin flakes (with particle size ~160 nm), and spherical zirconia particles (with particle size ~3 nm) (Figure 3). Boehmite flakes have rough dimpled surfaces, and the ZrO_2_ particles are attached to boehmite flakes. The mean particle size for the Al_2_O_3_–20 wt % ZrO_2_ sample is visibly smaller than that for the Al_2_O_3_–40 wt % ZrO_2_. Both phases—ZrO_2_ and boehmite—are uniformly distributed on the SEM and TEM images. Annealing at 1400 °C leads to a visible increase in the ZrO_2_ particle size (Figure 4). In addition, ZrO_2_ particles (spherical, dark particles) for both composition are isolated in the Al_2_O_3_ matrix and they are prevented from further growing (Figure 4c,d).

On the other hand, the results of the particle size distributions for ZrO_2_ phases (both t-ZrO_2_ and m-ZrO_2_) presented in Figure 5 show differences between compositions. The mean alumina particle size after sintering at 1400 °C was 480 nm for Al_2_O_3_–40 wt % ZrO_2_, and 750 nm for Al_2_O_3_–20 wt % ZrO_2_, while the average size of the t-ZrO_2_ grains based on TEM observations was 30 nm for both compositions. From TEM images it is also possible to infer that a and c lattice spacings are different in these grains, which confirms that their structure is tetragonal (Figure 4e).

As expected, the specific surface area (SSA_BET_) and density of the samples were affected by annealing at 600 °C and 1400 °C. The specific surface area for both compositions after annealing at 1400 °C was strikingly different compared to the values obtained after only annealing at 600 °C.

The nanoparticles’ size, size distribution, and morphology influence phase transformations in the alumina and zirconia composites. Furthermore, the phase transformation of Al_2_O_3_ influences the densification process. In the following paragraph, we will therefore discuss the sintering behavior of Al_2_O_3_–ZrO_2_ nanocomposites in relation to the phase composition.

Dilatometry results for Al_2_O_3_–20 wt % ZrO_2_ and Al_2_O_3_–40 wt% ZrO_2_ are presented in Figure 6. Other thermal properties of nanocomposites were previously reported in [56]. Three distinctive regions in dilatometry curves can be found:

- The first (1) refers to the temperature range before the nanocomposite will shrink (before onset temperature—Ton). This is the rearrangement region [57] which is visible from RT up to 1100 °C for Al_2_O_3_–20 wt % ZrO_2_, and up to 1150 °C for Al_2_O_3_–40 wt % ZrO_2_. Both of these values are approximately 100 °C lower than results published recently for a similar system by Scoton et al. [57].
- The second (2) step belongs to the shrinkage process, where the maximum shrinkage rates are found at 1208 °C for Al_2_O_3_–20 wt % ZrO_2_ and 1231 °C for Al_2_O_3_–40 wt % ZrO_2_. Both of these temperatures are also from 100 to 200 °C lower compared to relevant literature [57,58].
- The third (3) step distinguished on the dilatometry curves belongs to the end of the sintering process. The end of the sintering process for both compositions appears at approximately 1300 °C, and is associated with a 22% change of length.

It is important to analyze in detail the phase transformations for the ZrO_2_ nanoparticles in the temperature range from 1200 °C to 1400 °C, since nanoparticles sinter rapidly in this temperature region. The results of phase analysis for Al_2_O_3_–20 wt % ZrO_2_ and Al_2_O_3_–40 wt % ZrO_2_ nanopowders as a function of annealing temperature are shown in Figure 7. The phase content of as-synthesized powders was described before [12] and it is composed of fully crystalline boehmite, t-ZrO_2_, and m-ZrO_2_. Annealing at 600 °C leads to an AlO(OH)→ɣ-Al_2_O_3_ transformation. Subsequent annealing of Al_2_O_3_–20 wt % ZrO_2_ and Al_2_O_3_–40 wt % ZrO_2_ at 1200, 1400 and 1500 °C causes the following alumina transformation: ɣ-Al_2_O_3_→θ-Al_2_O_3_→α-Al_2_O_3_.

It is known that the specific temperatures for alumina transitions depend on the dopant concentration and alumina mean particle size [59]. In particular, the additives that reduce alumina particle growth during sintering and reduce nucleation sites α-Al_2_O_3_ (for example, ZrO_2_) [59] strongly influence phase transition temperatures. The second step of alumina nanoparticle transformation (θ-Al_2_O_3_→α-Al_2_O_3_) in nanocomposites is critical to understanding the sintering. For this reason, it is important to determine the temperature regions of stability of the θ-Al_2_O_3_ (theta alumina) phase. Sintering of nanosized Al_2_O_3_ powders accompanied by grain growth is explained by polymorphic phase transformations [60].

The effects of phase transformation on various Al_2_O_3_–ZrO_2_ composites sintering was already discussed in the literature [10,30,59,60,61,62]. For example, Chen et al. [10] found that shrinkage of Al_2_O_3_–ZrO_2_ composites is related to the θ→α-Al_2_O_3_ phase transformation taking place in the second step of sintering. During this stage, the coalescence of crystallites and necking were developed, which retarded densification as well as shrinkage. According to the authors [10], the final densification temperature is affected by the microstructure after the θ→α-Al_2_O_3_ phase transformation. The authors [10] found that the phase composition of the composite at various annealing temperatures depends on the aging time of the precipitated mixture obtained in the sol-gel process prior to sintering. They reported [10] that the phase transformation of θ-Al_2_O_3_ into α-Al_2_O_3_ can be shifted to temperatures higher than 1300 °C due to aging. Such phase transformation appears to be thanks to the transformation of nano-sized Al_2_O_3_ powder, and is connected to nucleation and growth processes. It was found that the shorter the aging time, the stronger the interaction between ZrO_2_ and Al_2_O_3_ particles [10]. Additionally, precipitated ZrO_2_ particles can prevent Al_2_O_3_ particles from contact which suppresses coalescence and delays θ→α phase transformation.

Lamouri et al. [59], who discussed transition of alumina phases and their influence on densification process, found that densification of alumina takes place due to nucleation and grain growth mechanisms. This process is influenced by grain size, starting nanoparticle morphology, dynamics of θ→α phase transformation in Al_2_O_3_, and the heating rate of the sintering process. The slow heating rate of the sintering process reduces the temperature of α-Al_2_O_3_ formation which is characterized by high activation energy [59].

In our case, θ-Al_2_O_3_ was observed in both materials even after annealing at temperatures higher than 1200 °C. According to recent publications [60,61,62], any grain growth process would lead to transformation of theta to alpha phase (α-Al_2_O_3_). In fact, Lamouri et al. [59] found that θ→α-Al_2_O_3_ phase transformation can happen only after θ-Al_2_O_3_ coarsens to a critical size (~20 nm). Such coarsening is underpinned by the Ostvald ripening process. The Ostvald ripening rate is determined by the probability of contact between two θ-Al_2_O_3_ particles and the material diffusion process. The precipitated zirconia particles can prevent θ-Al_2_O_3_ particles from coming into contact, thus suppressing the coalescence of θ-Al_2_O_3_ particles which in turn delays the θ→α-Al_2_O_3_ phase transformation. Such suppression of coalescence can be readily achieved in samples made by co-precipitation and quick crystallization in a microwave synthesis reactor.

Thus, above 1000 °C as a result of grain growth, only α-Al_2_O_3_ should be observed. It might be assumed that this process has not taken place during the present study because the addition of ZrO_2_ nanoparticles slows down the alumina particle growth. Rietveld refinement analysis of particle size shows that both compositions after annealing at 1400 °C are characterized by 30–80 nm t-ZrO_2_ and 50–200 nm m-ZrO_2_, while alumina particles are approximately 200 nm in diameter in both cases. In addition, ZrO_2_ nanoparticles remain within the alumina matrix grains as well as at the grain boundaries. The smaller ZrO_2_ particles seem to be entrapped within the alumina grains and they prevent alumina particles from growing (Figure 4 and Figure 5).

Equally, the nanoparticles’ morphology may also influence the transition temperatures between alumina phases. Levin et al. [60] studied phase transformation during annealing of boehmite and showed that the morphology of the starting materials has an influence on the θ→α-Al_2_O_3_ phase transformation. Lee et al. [61] showed that the platelet γ-Al_2_O_3_ did not transform to α-Al_2_O_3_, even after 1100 °C calcination. In our study, the calcination platelet morphology of boehmite was obtained, and no θ to α-Al_2_O_3_ transformation was observed up 1200 °C. Our results are in agreement with [61].

An analysis of results presented in Table 4 shows an agreement between nominal (predicted based on the composition of the precursors) and measured weight % of ZrO_2_ in all materials. The differences of ZrO_2_ wt % values measured by EDS and ICP-OES methods and by using Rietveld refinement are relatively minor. Our results show that the amount of t-ZrO_2_ phase in the case of Al_2_O_3_–20 wt % ZrO_2_ composite varies between 12.7 and 15.9 wt % in the whole temperature range. In the Al_2_O_3_–40 wt % ZrO_2_, t-ZrO_2_ varies between 8 and 25 wt % (Table 4). For the Al_2_O_3_–20 wt % ZrO_2_ composite, it was observed that amount of the t-ZrO_2_ phase was constant (within the experimental error) for the whole temperature range investigated (from 600 °C to 1400 °C). The weight ratio of t-ZrO_2_ to m-ZrO_2_ was 3.3:1. Only at the 1400 °C temperature was a decrease of t-ZrO_2_/m-ZrO_2_ observed. It is interesting to note that the t-ZrO_2_/m-ZrO_2_ ratio after sintering up to 1200 °C was very close to the one observed for the composites of Al_2_O_3_–3 mol %YSZ presented in [30].

As far as the Al_2_O_3_–40 wt % ZrO_2_ material is concerned, a different behavior was observed in comparison to the Al_2_O_3_–20 wt % ZrO_2_. The t-ZrO_2_ phase was dominant only after 600 °C annealing. Further annealing causes a systematic increase of the m-ZrO_2_ phase content. This confirms that Al_2_O_3_–40 wt % ZrO_2_ has lower thermal stability, and isolation of t-ZrO_2_ in the Al_2_O_3_ matrix is weaker.

It was found that thermal expansion coefficients for Al_2_O_3_–40 wt % ZrO_2_ vary due to phase transition. Contrary to the Al_2_O_3_–40 wt % ZrO_2_ composition, Al_2_O_3_–20 wt % ZrO_2_ is characterized by only one value of thermal expansion coefficient. The thermal expansion coefficient for Al_2_O_3_–20 wt % ZrO_2_ is 10.29 × 10^−6^ K^−1^, while those for Al_2_O_3_–40 wt % ZrO_2_ are 8.99 and 13.84 × 10^−6^ K^−1^ (Table 7). This experiment confirms that Al_2_O_3_–20 wt % ZrO_2_ nanocomposite is not characterized by martensitic transformation (t↔m ZrO_2_), which is beneficial for mechanical properties. For Al_2_O_3_–20 wt % ZrO_2_ it was found that the t-ZrO_2_ phase was stable up to 1400 °C, which is also attributed to nanoparticle separation.

## 5. Conclusions

In this paper, we showed a new method for the manufacturing of Al_2_O_3_–ZrO_2_ nanocomposites with reduced sintering temperature. The sintering temperatures were found to be 1209 °C for Al_2_O_3_–20 wt % ZrO_2_ and 1231 °C for Al_2_O_3_–40 wt % ZrO_2_ nanocomposites. These temperatures were approximately 100 °C lower than previously reported for similar compositions. In the above nanostructures, the θ-Al_2_O_3_ nanoparticles are surrounded by small zirconia nanoparticles, which prevent their growth and stabilize the θ-phase up to 1200 °C. The reason for the decrease of the sintering temperature is related to:

- Uniform distribution of zirconia nanoparticles within the alumina matrix;
- Maintaining up to relatively high temperature a grain size in the nano range. As it is known, the driving force for sintering nanomaterials is higher than that for microcrystals;
- θ-Al_2_O_3_ stability was extended up to 1200 °C, with enhancement of that phase’s sintering.

For the Al_2_O_3_–20 wt % ZrO_2_ composition, we observed stability of the zirconia tetragonal phase up to 1400 °C. We associate such stability with the mutual separation of zirconia nanoparticles in the alumina matrix. A different behavior was observed for Al_2_O_3_–40 wt % ZrO_2_. The t-ZrO_2_ phase was dominant only after 600 °C annealing. Further annealing causes a systematic increase of the m-ZrO_2_ phase content. This confirms that Al_2_O_3_–40 wt % ZrO_2_ has lower thermal stability, and isolation of t-ZrO_2_ in the Al_2_O_3_ matrix is weaker.

## Figures and Tables

**Table 1:** Characterization of FTIR-ATR bands in Al_2_O_3_–(20,40 wt %) ZrO_2_ samples.

**ZrO_2_ Bands in As-Synthesized Samples (cm^−1^)**	**Literature**	**AlO(OH) Bands in As-Synthesized Samples (cm^−1^)**	**Literature**
		472, 605	Bands between 780 and 500 cm^−1^ can be assigned to the vibration mode of AlO_6_ [36]
		737	Al–O–Al framework [37]
		900	Al–O band stretching vibration of boehmite [10]
		1070, 1170	(HO)–Al=O asymmetric stretching and the O–H bending, respectively [46]
1338	Bending vibration of Zr–OH groups [37], and it might be t-ZrO_2_ [48]	1170	Al–O–H vibrations [48]
1566	t-ZrO_2_ [48]	1668	O–H bending mode [37]
3357	Stretching vibration of hydroxyl group and the interlayer water molecules [40]	3099, 3311	Asymmetric and symmetric O–H stretching vibrations from (O)Al–OH [37]
**ZrO_2_ Bands in Annealed Samples (cm^−1^)**	**Literature**	**Al_2_O_3_ Bands in Annealed Samples (cm^−1^)**	**Literature**
440	symmetric Zr–O–Zr stretching mode related with t-ZrO_2_ phase [46] It might me ν(Zr–O) band from t-ZrO_2_ [51]	447	Al–O stretching mode in α-Al_2_O_3_ [47]
484	t-ZrO_2_ [52], stretching vibrations of Zr–O in ZrO_2_	495	α-Al_2_O_3_ [47]
567 719	Zr–O stretching vibrations [50]	595	Al–O stretching mode in octahedral structure [50]
		642	asymmetric Zr–O–Zr stretching mode from the m-ZrO_2_ phase [49]

**Table 2:** Raman spectral data for Al_2_O_3_–(40,20 wt %) ZrO_2_ after annealing at 600 °C and 1400 °C [45,52,53,54,55].

Al_2_O_3_–(20,40 wt %) ZrO_2_ after Annealing at 600 °C	Wavenumbers (cm^−1^)	Literature	Al_2_O_3_–(20,40 wt %) ZrO_2_ after Annealing at 1400 °C	Wavenumbers (cm^−1^)	Literature
			Al_2_O_3_–(20 wt %) ZrO_2_ after annealing at 1400 °C		
t-ZrO_2_	148, 265, 312	[49]	t-ZrO_2_	147, 270, 315, 459	[49]
m-ZrO_2_	177, 188, 215, 355, 382, 477, 513, 559, 637	[49]	m-ZrO_2_	179, 190, 381, 419, 578, 647	[49]
γ-Al_2_O_3_	234, 404	[52,53]	α-Al_2_O_3_	381, 419, 431,578,647, 751	[52,53,54,55]
			Al_2_O_3_–(40 wt %) ZrO_2_ after annealing at 1400 °C		
			t-ZrO_2_	145,269, 314, 456	[49]
			m-ZrO_2_	179, 190, 336, 351, 380, 417, 477, 507, 598, 647	[49]
			α-Al_2_O_3_	380, 417, 647, 753	[52,53,54,55]

**Table 3:** Comparison of specific surface area (SSA_BET_), helium density, and particle size for Al_2_O_3_–(20,40 wt %) ZrO_2_ nanocomposites annealed at 600 °C and 1400 °C.

Composition (wt %)	Thermal Treatment	Specific Surface Area SSA_BET_ (m^2^/g)	Helium Density (g/cm^3^)	Densification (%)
Al_2_O_3_–20% ZrO_2_	600 °C	177.2097 ± 0.6228	3.5442 ± 0.0062	84
	1400 °C	3.3659 ± 0.0085	4.2073 ± 0.0087	
Al_2_O_3_–40% ZrO_2_	600 °C	95.9208 ± 0.4215	3.8257 ± 0.0455	90
	1400 °C	0.5900 ± 0.0042	4.2260 ± 0.0133	

**Table 4:** Chemical and phase composition of Al_2_O_3_–(20,40 wt %) ZrO_2_ nanocomposites annealed in the 600–1400 °C temperature range.

Composition (wt %)	EDS (wt % of ZrO_2_)	ICP-OES (wt % of ZrO_2_)	Phase Composition Obtained from Rietveld Refinement for Samples after Annealing at Different Temperatures (wt %)		
			600 °C	1200 °C	1400 °C
Al_2_O_3_–20% ZrO_2_	20.14 ± 1.37	21 ± 1.09	83.3 ɣ −Al_2_O_3_	82.3 θ−Al_2_O_3_	80.5 α−Al_2_O_3_
			12.9 t−ZrO_2_	14.5 t−ZrO_2_	12.6 t−ZrO_2_
			3.8 m−ZrO_2_	3.2 m−ZrO_2_	6.8 m−ZrO_2_
Al_2_O_3_–40% ZrO_2_	38.47 ± 2.61	40 ± 0.81	67.4 ɣ −Al_2_O_3_	61.3 θ −Al_2_O_3_	59.4 α−Al_2_O_3_
			25.9 t−ZrO_2_	13.8 t−ZrO_2_	8.0 t−ZrO_2_
			6.7 m−ZrO_2_	24.9 m−ZrO_2_	32.6 m−ZrO_2_

**Table 5:** Atom position in θ-Al_2_O_3_, α-Al_2_O_3_, m-ZrO_2_, and t-ZrO_2_ based on Rietveld refinement.

Phase: θ-Al_2_O_3_, C 2/m				Phase: m-ZrO_2_, P 21/c			
**Atom Position**	**x**	**y**	**z**	**Atom Position**	**x**	**y**	**z**
Al1:Al^3+^	0.10130	0.00000	0.79440	Zr:Zr^4+^	0.27605	0.03987	0.20927
Al2:Al^3+^	0.35230	0.00000	0.68740	O1:O^2−^	0.06633	0.33052	0.34468
O1:O^2−^	0.16270	0.00000	0.12280	O2:O^2−^	0.45232	0.75875	0.47537
O2:O^2−^	0.48950	0.00000	0.26130				
O3:O^2−^	0.82990	0.00000	0.43860				
**Phase: α-Al_2_O_3_, 167**	**x**	**y**	**z**	**Phase: t-ZrO_2_, P 42/nmc**	**x**	**y**	**z**
Al:Al^3+^	0.00000	0.00000	0.35230	Zr:Zr^4+^	0.75000	0.25000	0.75000
O:O^2−^	0.30640	0.00000	0.25000	O:O^2−^	0.25000	0.25000	0.55000

**Table 6:** Cell parameters of θ-Al_2_O_3_, α-Al_2_O_3_, m-ZrO_2_, and t-ZrO_2_ phases calculated in Rietveld refinement for Al_2_O_3_–20 wt % ZrO_2_ and Al_2_O_3_–40 wt % ZrO_2_ nanocomposites annealed at 1200 °C and 1400 °C.

**Unit Cell Dimensions in Al_2_O_3_–20 wt %ZrO_2_**							
**T = 1200 °C**				**T = 1400 °C**			
**Phase:**	**t-ZrO_2_**			**Phase:**	**t-ZrO_2_**		
a (Å)	b (Å)	c (Å)	α,β,γ (^o^)	a (Å)	b (Å)	c (Å)	α, β, γ (^o^)
3.597020	3.597020	5.191563	α = β = γ 90.0000	3.598789	3.598789	5.200136	α = β = γ 90.0000
**Phase:**	**m-ZrO_2_**			**Phase:**	**m-ZrO_2_**		
5.093851	5.141055	5.313514	α = γ; β 90.0000; 100.5483	5.149243	5.186935	5.318353	α = γ; β 90.0000; 99.0302
**Phase:**	**θ-Al_2_O_3_**			**Phase:**	**α-Al_2_O_3_**		
11.802021	2.911568	5.622274	α = γ; β 90.0000; 104.0478	4.760565	4.760565	12.996780	α = β; γ 90.0000; 120.0000
**Unit Cell Dimensions in Al_2_O_3_–40 wt %ZrO_2_**							
**T = 1200 °C**				**T = 1400 °C**			
**Phase:**	**t-ZrO_2_**			**Phase:**	**t-ZrO_2_**		
a (Å)	b (Å)	c (Å)	α,β,γ (^o^)	a (Å)	b (Å)	c (Å)	α,β,γ (^o^)
3.597343	3.597343	5.194126	α = β = γ 90.0000	3.599882	3.599882	5.200407	α = β = γ 90.0000
**Phase:**	**m-ZrO_2_**			**Phase:**	**m-ZrO_2_**		
5.146611	5.188430	5.310700	α = γ; β 90.0000; 98.9165	5.152256	5.190232	5.315270	α = γ; β 90.0000; 99.0657
**Phase:**	**θ-Al_2_O_3_**			**Phase:**	**α-Al_2_O_3_**		
11.795719	2.911480	5.620936	α = γ; β 90.0000; 104.0352	4.762267	4.762267	12.999507	α = γ; β 90.0000; 120.0000

**Table 7:** Thermal properties for Al_2_O_3_–(20,40 wt %) ZrO_2_ nanocomposites obtained from dilatometry experiments.

**Composition (wt %)**	**Sintering Temperature (°C)**	**Thermal Expansion Coefficient ^1^ (10^−6^ K^−1^)/Structure**		**Transition Temperature (°C)**
		**t-ZrO_2_**	**m-ZrO_2_**	
Al_2_O_3_–20% ZrO_2_	1209	10.29	N/A	N/A
Al_2_O_3_–40% ZrO_2_	1231	8,99	13,84	1153

^1^ Thermal expansion coefficients with corresponding transition temperatures obtained for nanocomposites sintered at 1500 °C.
